# Low-Energy Electron and Positron Scattering by Lysine: Cross Sections and Theoretical Insights into Possible DEA Pathways

**DOI:** 10.1021/acs.jpca.5c05601

**Published:** 2025-10-02

**Authors:** Cesar A. do Amaral, Raul V. B. Morás, Giseli M. Moreira, Sergio d’Almeida Sanchez, Alessandra Souza Barbosa

**Affiliations:** † Departamento de Física, 425918 Universidade Federal de Santa Catarina, Florianópolis, SC 88040-900, Brazil; ‡ Departamento de Física, 245075 Universidade Federal do Paraná, Curitiba, PR 81531-980, Brazil; § Departamento de Física Aplicada, Universidade Federal da Integração Latino-Americana, Foz do Iguaçu, PR 85867-970, Brazil; ∥ Departamento de Física, 28122 Universidade Estadual do Centro-Oeste, Guarapuava, PR 85040-080, Brazil

## Abstract

We report a theoretical investigation of low-energy electron and positron scattering by the lysine molecule. The calculations were performed using the Schwinger multichannel method with different levels of approximation for each projectile. The static-exchange (SE) and static-exchange plus polarization (SEP) approximations were used for electrons, while the static plus polarization (SP) approximation was used for positrons. Our results for electron scattering show a π* resonance centered at 3.95 eV for SE and 2.73 eV for SEP in the integral cross section, as well as a large structure around 11.0 eV for SE and 9.0 eV for SEP, which may be associated with overlapping σ* resonances. For comparison purposes, since there are no theoretical or experimental cross sections available in the literature, a semiempirical relation was employed to estimate the value of the π* resonance. We also compared the results obtained for electron and positron scattering, showing similar behavior at very low energy due to the dipole interaction and approximately the same order of magnitude from 2 to 6 eV. Differential cross sections for both projectiles also exhibit the same dominant wave pattern. To investigate the connection between the resonance and the dissociative electron attachment (DEA), we calculated threshold energies for hydrogen loss from different sites in the molecule, identifying a low-energy channel (1.85 eV) consistent with previous DEA studies on similar systems. Furthermore, excited electronic states of lysine were obtained by using time-dependent density functional theory (TDDFT), providing additional insight into possible Feshbach-type DEA pathways. These results represent the first theoretical study of scattering processes involving electrons and positrons with lysine and offer a foundation for future experimental and computational investigations.

## Graphical abstract

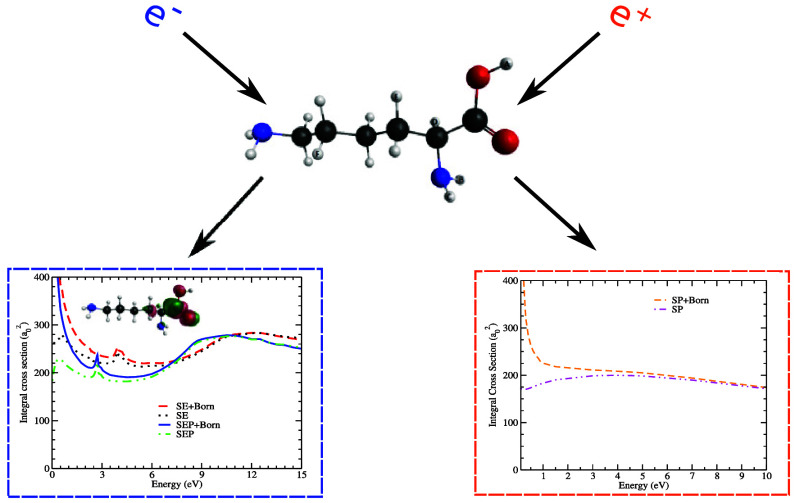

## Introduction

Low-energy electron and positron scattering is an essential area of research across multiple fields. In recent years, the significance of these studies in biology has become increasingly apparent. When ionizing radiation interacts with cells, secondary low-energy electrons are generated in great numbers, typically with energies up to 10 eV. These electrons can collide with various molecules, including the constituents of DNA, causing single- and double-strand breaks via a process known as Dissociative Electron Attachment (DEA).^1^ DEA is initiated by the capture of the incident electron in a resonant state, followed by energy transfer to the nuclear degrees of freedom and subsequent breakage of a molecular bond.

In the case of a positron as a projectile, understanding the path taken by it in the human body is key in improving the positron emission tomography (PET).^2^ In the human body, the positron undergoes several collision processes along its track until it is thermalized and annihilated with an electron in the medium. The two γ rays, due to the annihilation, are detected in coincidence by the apparatus that is responsible for reconstructing the diagnostic image. Hence, different methodologies have been created to calculate positron tracks in the human body,^3,4^ all requiring positron scattering cross sections as input data.

Comparing cross sections between electrons and positrons is of fundamental interest as one is the antiparticle of the other. The differences among the projectiles arise not only due to the sign of the Coulomb interaction potential but also due to the absence of the exchange potential for positrons, besides the possibilities of annihilation and positronium formation. Therefore, to better understand the dynamics of the collision of electrons and positrons with the same molecular target, several studies have been developed (see for instance^5–9^ ), which point out the main differences and similarities between the cross sections of the two projectiles. However, these studies have mostly focused on relatively small molecules. This work aims to analyze positron and electron scattering by lysine, which consists of 24 atoms. Despite the significant challenge posed by the size of this molecule, its importance makes it a crucial target for investigation.

Lysine (C_6_H_14_N_2_O_2_, see Figure 1), an amino acid found in the human body, plays an essential role in various biological processes. It has been shown to decrease the replication of the herpes virus,^10^ reduce chronic stress and anxiety levels,^11,12^ strengthen the immune system,^13^ and potentially have a positive effect on the treatment of cataracts when used as the lysine salt of bendazac.^14^ Lysine is also being studied for the treatment of pancreatic cancer^15^ and for the recognition of DNA damage sites by lysine conjugates.^16^ In a recent study conducted by Verma and co-workers,^17^ the role of four amino acids on low-energy electron attachment to DNA was investigated, and among them is the lysine molecule. In this work, the authors used computational simulations considering the cytosine molecule within the bulk of amino acids. Additionally, lysine exhibits optical isomerism, meaning it can exist in both dextrorotary (d-lysine) and levorotary (l-lysine) forms as well as several other conformers. However, no difference is expected between such isomers in the context of the scattering calculation since the physicochemical properties are the same for optical isomers with only the direction in which polarized light is deflected being different.

**Figure 1:**
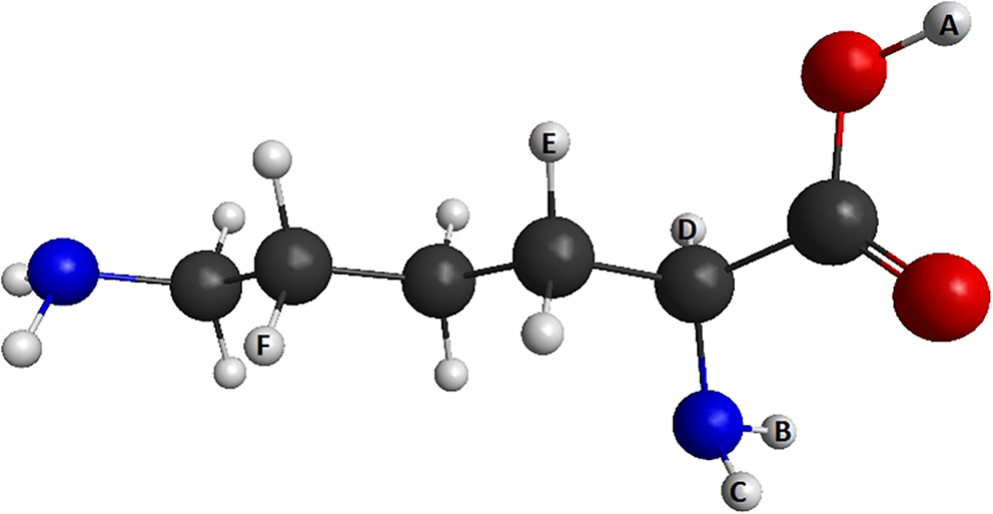
Geometry of the most stable conformer of the lysine molecule (LYS12). Black centers are carbon atoms, blue centers are nitrogen atoms, red centers are oxygen atoms, and white centers are hydrogen. The indexes A, B, C, D, and F in the hydrogen atoms will be discussed in the Results and Discussion section (generated with MacMolPlt^18^).

This paper reports on the calculation of integral and differential cross sections for electron and positron scattering with the lysine molecule using the Schwinger multichannel (SMC) method. Calculated data for the cross section can be found at[Fn fn1]. The calculations were carried out at different levels of approximation, including static-exchange and static-exchange plus polarization for electrons and static plus polarization for positrons. The Born-closure correction was also considered to account for the long-range dipole moment of the system. We also employed a semiempirical relation to estimate the vertical attachment energy (VAE), given the absence of theoretical or experimental data in the literature for comparison. Additionally, we conducted calculations to estimate energy thresholds for H loss, and we also obtained the energies and assignments of the first few excited states, which can play a role in the low-energy positron and electron scattering collisions with this system. Moreover, we used experimental work on electron interactions with other amino acids as reference, such as glycine, alanine, proline, phenylalanine, valine, and leucine, for comparison. These studies have shown that these systems support a π* resonance around 1–2 eV and exhibit a sharp H-loss channel at the same energy range.^19–23^

Beyond the analysis of scattering cross sections and DEA thresholds, we also investigated the electronic excited states of lysine using time-dependent density functional theory (TDDFT). This was done to explore the possibility of alternative DEA mechanisms mediated by Feshbach or core-excited resonances, which are relevant in the context of radiation-induced damage in biomolecules. The proximity between some excited states and the dissociation thresholds suggests a potential role for these states in electron-induced fragmentation, complementing the picture provided by the π* resonances.

## Theory and Computational Details

We used the Schwinger multichannel (SMC) method, implemented with norm-conserving pseudopotentials (SMCPP)^24,25^ for electron scattering and the SMC method for positron scattering,^26^ to obtain integral and differential cross sections. Since these methods have been described in detail elsewhere, we provide a brief overview in this paper.

For both projectiles, the working expression to obtain the scattering amplitude is given by,

1 $$f\left({\mathrm{k⃗}}_{i},{\mathrm{k⃗}}_{f}\right)=-\frac{1}{2\pi }\sum_{m,n}\left\langle S_{{\mathrm{k⃗}}_{f}}\right|V\left|\chi_{m}\right\rangle {\left(d^{-1}\right)}_{\mathrm{mn}}\left\langle \chi_{n}\right|V\left|S_{{\mathrm{k⃗}}_{i}}\right\rangle$$

in which the *d*_*mn*_ matrix elements are given by

2 $$d_{\mathrm{mn}}=\left\langle \chi_{m}\right|A^{\left(+\right)}\left|\chi_{n}\right\rangle$$

The *A* ^(+)^ operator, for electron scattering, is given by

3 $$A^{\left(+\right)}=\frac{1}{2}\left(\mathrm{PV}+\mathrm{VP}\right)-VG_{p}^{\left(+\right)}V+\frac{Ĥ}{N+1}-\frac{1}{2}\left(ĤP+PĤ\right)$$

and, for positron scattering, by

4 $$A^{\left(+\right)}=\mathrm{PVP}+QĤQ-VG_{P}^{\left(+\right)}V$$

In the above expressions, the χ_*m*_ terms represent trial configuration-state functions (CSFs) of the (*N*+1) -electron system, while *V* denotes the interaction potential between the incident particle and the target nuclei. The open channel space projector is represented by *P*, and *G*_*P*_^(+)^ denotes the free-particle Green’s function that is projected onto the *P*-space. The difference between the total collision energy and the (*N*+1) -electron Hamiltonian in the fixed nuclei approximation (*H* = *H*_0_+ *V*) is represented by *Ĥ*. The notation |*S*_*k⃗*_*i*,*f*__⟩ represents the solution of unperturbed Hamiltonian *H*_0_, which is the product of a target state and a plane wave. In the case of positron scattering, *Q* represents the projector on closed electronic channels of the target.

Electrons and positrons scattering differ not only in the sign of the potential but also in the fact that the positron is distinguishable from the electrons of the molecular target. Furthermore, the positron can annihilate with one of the electrons of the target and form positronium, though this will not be dealt with in this work, as positronium formation constitutes an inelastic channel that is not included in the present SMC calculations. This suggests that our cross sections above the threshold of the positronium formation channel may be overestimated. To perform these calculations, distinct approximations were used for each scattering process. For electron scattering, the static-exchange (SE) and static-exchange plus polarization (SEP) levels of approximations were employed. The SE approximation considers the exchange effects between the incident electron and the electrons of the target; however, it assumes that the electronic cloud remains frozen throughout the collision process. On the other hand, the SEP considers the distortion of the electronic cloud due to the incoming electron. For positron scattering, only the static plus polarization (SP) approach was used.

The CSFs in the SE approximation are constructed using the target ground state (|Φ_0_⟩), which was obtained at the Hartree–Fock level, and a single-particle function (|ϕ_*m*_⟩):

5 $$\left|\chi_{m}\right\rangle =A_{N+1}\left[\left|\Phi_{0}\right\rangle ⊗\left|\phi_{m}\right\rangle \right]$$

where *A*_*N*+1_ is the antisymmetrization operator of *N*+1 electrons.

In the SEP approximation, we include the polarization effects through virtual single excitations of the molecular target by adding to eq 5 CSFs of the type:

6 $$\left|\chi_{\mathrm{im}}\right\rangle =A_{N+1}\left[\left|\Phi_{i}\right\rangle ⊗\left|\phi_{m}\right\rangle \right]$$

where the state |Φ_*i*_⟩ refers to *N*– electron Slater determinants obtained by virtual excitations from the occupied (hole) orbitals to a set of unoccupied (particle) orbitals. For the SP approximation, the configuration space is constructed as in eq 5 plus eq 6 but without the antisymmetrization operator.

To perform our calculations, we first optimized the ground-state geometry using the GAMESS computer package^27^ with second-order Mø ller-Plesset perturbation theory (MP2) and the aug-cc-pVDZ basis set. Since there are different conformers and the computational cost for this large molecule is too expensive, we used only the most abundant equilibrium geometry at room temperature, LYS12, according to ref 28. Geometry optimization and all scattering calculations were carried out within the *C*_1_ point group.

To represent the molecular target in electron scattering, we replaced the core electrons of carbon, oxygen, and nitrogen atoms using the norm-conserving pseudopotentials of Bachelet, Hamann, and Schlüter (BHS).^29^ For the valence electrons, we used an uncontracted 5*s*5*p*3*d* basis set for oxygen^30^ and an uncontracted 6*s*5*p*1*d* basis set for carbon and nitrogen.^31^ For hydrogen, we used the 4*s*/3*s* basis set^32^ with an added function *p* with an exponent of 0.75. For positron scattering calculations, we used the DZV++(2*p*,1*d*) basis set. Here, we remember that, for electrons, the generated basis is designed for the use of pseudopotentials, where the indicated basis describes the target’s valence electrons, while the core electrons are described by the BHS potentials. Note that this can be achieved because the core electrons are inaccessible to low-energy incident electrons due to electronic repulsion. For positrons, these core electrons are accessible by electronic attraction, requiring a basis that describes them, such as the indicated basis. In the SE calculations, we used Hartree–Fock canonical virtual orbitals. In the SEP and SP approaches, we used the Modified Virtual Orbitals^33^ (MVOs) to represent the particle and scattering orbitals. The MVOs were generated by diagonalizing a + 6 cationic Fock operator and were made orthogonal to the occupied orbitals. For the SEP approximation, we constructed the closed-channel space with singlet- and triplet-coupled excitations. We constructed the virtual excitations from 30 valence orbitals to the 23 lowest MVOs, which were used as scattering orbitals, resulting in 16,202 CSFs. For the SP approach, we used the 30 last occupied as hole, 11 first unoccupied orbitals as particle, and 51 scattering orbitals, resulting in 17,064 CSFs.

Lysine is a polar molecule, and the calculated dipole moment in this work was 1.11 D, in agreement with the calculated value of 1.09 D, obtained using the MP2/6–31++G** basis set.^28^ Since the SMC method employed in this study uses square integrable functions (*L* ^2^) in the expansion of the scattering wave function, we applied the Born-closure^34^ procedure to account for the long-range character of the dipole potential through the first Born approximation (FBA). The Born-closure scattering amplitude is expressed as follows:

7 $$f\left({\mathrm{k⃗}}_{i},{\mathrm{k⃗}}_{f}\right)=f^{\mathrm{FBA}}\left({\mathrm{k⃗}}_{i},{\mathrm{k⃗}}_{f}\right)+\sum_{l=0}^{l_{\max}}\sum_{m=-l}^{l}\left[f_{\mathrm{lm}}^{\mathrm{SMC}}\left({\mathrm{k⃗}}_{i},{\mathrm{k⃗}}_{f}\right)-f_{\mathrm{lm}}^{\mathrm{FBA}}\left({\mathrm{k⃗}}_{i},{\mathrm{k⃗}}_{f}\right)\right]Y_{l,}^{*}\left({\mathrm{k̂}}_{f}\right)$$

where the dipole potential scattering amplitude obtained within the FBA is denoted as *f* ^FBA^. The outgoing angular dependence of SMC’s scattering amplitude is expanded in spherical harmonics to obtain *f*_*lm*_^SMC^(*k⃗*_*i*_,*k⃗*_*f*_), while the outgoing angular dependence of FBA’s scattering amplitude is expanded in spherical harmonics to obtain *f*_*lm*_^FBA^(*k⃗*_*i*_,*k⃗*_*f*_). To compute the differential cross sections with the Born-closure procedure, we chose a certain value for *l*_max_ so that the DCS calculated with the SMC and FBA agree after a certain angle. Lower partial waves of the scattering amplitude, up to *l*_max_, are described within the SMC method, while higher partial waves, from *l*_max_ + 1 to +∞, are described with the FBA for the dipole potential. The values of *l*_max_ chosen in both the SE and SEP approximations for lysine can be seen in Table 1. For positron scattering, the values of *l*_max_ chosen can be seen in Table 2.

**Table 1:** *l*_max_ Chosen for Lysine in Order to Take Into Account the Long-Range Effect of the Permanent Dipole of the Target Molecule for SE and SEP^a^

*l*_max_	SE	SEP
2	*E* ≤ 0.1 eV	*E* ≤ 0.1 eV
3	0.1 eV < *E* ≤ 0.6 eV	0.1 eV < *E* ≤ 0.4 eV
4	0.6 eV < *E* ≤ 1.0 eV	0.4 eV < *E* ≤ 1.0 eV
5	1.0 eV < *E* ≤ 1.6 eV	1.5 eV < *E* ≤ 1.25 eV
6	1.6 eV < *E* ≤ 3.6 eV	1.25 eV < *E* ≤ 3.25 eV
7	3.6 eV < *E* ≤ 4.5 eV	3.25 eV < *E* ≤ 4.0 eV
8	4.5 eV < *E* ≤ 6.5 eV	4.0 eV < *E* ≤ 6.5 eV
9	6.5 eV < *E* ≤ 9.0 eV	6.5 eV < *E* ≤ 7.25 eV
10	10.0 eV < *E*	7.25 eV < *E*

a *E* is the electron’s incident energy.

**Table 2:** *l*_max_ Chosen for Lysine, in Order to Take Into Account the Long-Range Effect of the Permanent Dipole of the Target Molecule for SP^a^

*l*_max_	SP
3	*E* ≤ 0.2 eV
4	0.2 eV < *E* ≤ 0.8 eV
5	0.8 eV < *E* ≤ 1.0 eV
6	1.0 eV < *E* ≤ 2.0 eV
7	2.0 eV < *E* ≤ 3.5 eV
8	3.5 eV < *E* ≤ 5.0 eV
9	5.0 eV < *E* ≤ 8.0 eV
10	8.0 eV < *E*

a *E* is the positron’s incident energy.

The scattering Hamiltonian (*H*_*N*+1_) was diagonalized in order to find the resonant states of the *N*+1 electron system. The resonance assignment was then performed by identifying the eigenvalue closest to the resonance feature observed in the cross section and analyzing the corresponding orbital associated with this eigenvalue. Furthermore, the single-particle orbital was built from the eigenstate of *H*_*N*+1_, whose eigenvalue is close to the energy of the resonant structure appearing in the cross section. Such an orbital was constructed according to the equation

8 $$\left|\phi_{j}\right\rangle =\sum_{m}^{n_{\mathrm{sc}}}\left|\varphi_{m}\right\rangle \left\langle \chi_{m}\right|\Psi_{j}^{N+1}\rangle$$

where the sum runs over all (*n*_*sc*_) CSFs that belong to the SE space (|χ_*m*_⟩), |φ_*m*_⟩ is the scattering orbital employed in the construction of |χ_*m*_ ⟩, and |Ψ_*j*_^*N*+1^⟩ is the *H*_*N*+1_ eigenvector.

## Results and Discussion

### Electron Scattering

To estimate the vertical attachment energy (VAE), we used a semiempirical scaling relation based on the Koopmans’ theorem.^35^ The VAE is an estimated value to the position of resonances, and to do this, we followed the same procedure proposed by Aflatooni et al.:^35^ we optimized the ground-state geometry of the molecule with the 6–31G­(*d*) basis set and then calculated the virtual orbital energy (VOE) in the optimized geometry. This was done using the GAMESS software.^27^ By comparing the calculated VOE and measured VAE, the authors found that VAE = (VOE – 2.5553)/1.3749,^35^ in units of eV. It should be noted that this semiempirical scaling relation is an estimative. Previous studies have shown that VAE estimates can over- or underestimating resonance positions, when compared to scattering calculations or experiments.^36^ Nevertheless, this procedure has been validated across a broad set of amino acids and similar systems, and thus provides a reliable guideline in the absence of direct experimental data.^37^ In our analysis, we use the VAE as a reference baseline against which SE and SEP results are compared, and due to computational limitations that prevent us from calculating lysine with a larger configuration space, we also use it as a reference value for discussions involving DEA pathways.

The most stable conformation of the lysine exhibits an approximate population abundance of 15% at room temperature; hence, we have also opted to calculate the VAE for all other conformers exceeding a 9% abundance threshold.^28^ These values are displayed in Table 3, revealing that there is no appreciable difference and are close to all other similar amino acids.^19–23^

**Table 3:** Estimated π* Resonance Position for More Abundant Conformations of Lysine

conformation	abundance (%)	VAE (eV)
LYS12	14.9	1.65
LYS13	10.8	1.58
LYS14	13	1.55
LYS15	9.7	1.53
LYS17	9.4	1.67

Recently, Ribas et al.^38^ have shown for glycine, the smallest amino acid, that although the resonant structures for different conformers are located in the same energy range, the magnitude of the integral cross sections is different. Their differential cross section analysis further indicated that, at lower impact energies, the cross sections are more sensitive to the different spatial distributions of atoms in each conformer, while with increasing energies, these discrepancies are reduced, especially for conformers with large dipole moments. Due to the size of the lysine molecule, however, a similar systematic study is not feasible here. We therefore calculated the cross sections exclusively for the conformer with the greatest abundance, LYS12. For lysine, we expect that the calculated resonance energies are still representative, while the absolute magnitudes of the cross sections may be somewhat sensitive to conformational averaging.

Figure 2 shows the integral cross sections obtained for electron scattering in the SE and SEP approximations with and without the Born-closure procedure. The π* resonance, which appears at 3.95 eV in the SE results and at 2.73 eV in the SEP results, is clearly visible in the figure. Additionally, a large structure between 10 and 15 eV is observed, possibly generated by an overlap of σ* resonances. The positions of the π* resonance and the center of the overlap of σ* resonances are summarized in Table 4. For comparison purposes, we also include the VAE results in this table. Furthermore, in the same Figure 2, it is possible to notice that the influence of the Born approximation is significant for energies below 7 eV and does not alter the position of the resonance, as expected.

**Figure 2:**
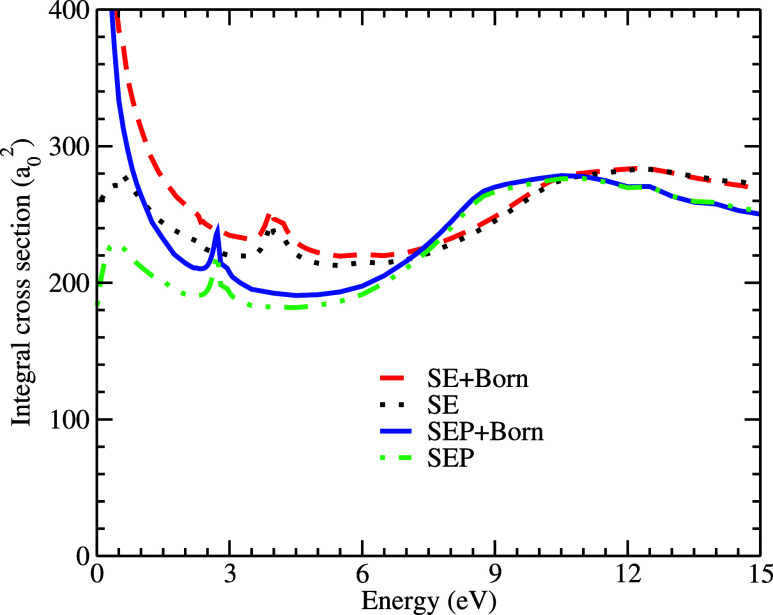
Elastic integral cross sections for electron scattering with the lysine molecule. We compare our SE and SEP, both with and without the Born-closure procedure. See the text for the further discussion.

**Table 4:** Position of the π* Shape-Resonance and the Center of σ* Resonances Overlapped Computed in the SE and SEP Approximations^a^

configuration	π*	σ*
SE	3.95	12.2
SEP	2.73	10.5
VAE	1.65	

a We also show the estimation of the VAE. All values are in eV.

As there are no other theoretical or experimental data available for this molecule, the discussion of our results is based on the value obtained via VAE. This value is considerably smaller than ours, even with the inclusion of polarization effects. We emphasize, however, that the VAE is not intended to provide an exact prediction but rather a reference guide, as discussed in the literature.^36,37^ The discrepancy observed here is probably due to the computational limits imposed by lysine being a large molecule and belonging to the *C*_1_ group, which prevents the use of symmetry to facilitate our calculations. Importantly, when polarization effects are included (SE compared with SEP), the calculated resonance shifts toward the VAE value. This suggests that with further improvement of the scattering calculation, such as expanding the configuration space, the agreement with the VAE could be even closer. Nevertheless, the VAE is close to what has been previously obtained in similar systems,^19–23^ which supports its validity as a comparative baseline. Because of these, even though the method is not exact, the results cited and those in the literature led us to use the VAE as a basis for our discussions. Furthermore, as we will see in the DEA Thresholds for Hydrogen Loss Section, the H-loss values differ sufficiently that, even if the VAE value is not exact, we can discuss the importance of this π* resonance in the DEA threshold.

Figure 3 shows the differential cross sections for electron scattering with and without the Born-closure approximation. As expected, there is a noticeable difference between the DCSs at low angles, i.e., below 15°, due to the inclusion of the long-range dipole interaction. At 3 eV, we observed a minimum in the cross sections, which correspond to a major contribution from a *p*-type wave pattern. At 5 eV, the DCSs show two minima, which are associated with a dominant *d*-type wave pattern. Finally, an *f*-type wave dominant pattern was noticeable at 7 and 10 eV, since the DCSs exhibit three minima.

**Figure 3:**
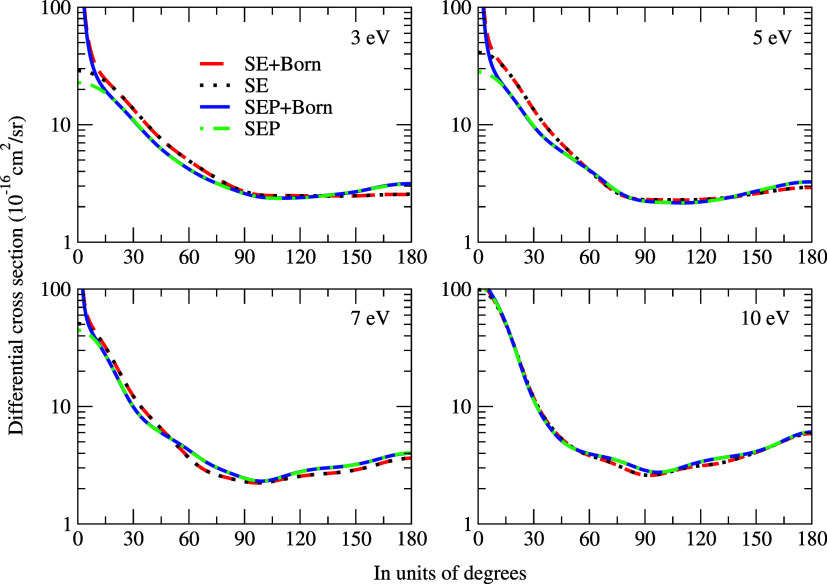
Differential cross sections for electron scattering in the lysine molecule. We compare our results obtained at SE and SEP approximations, with and without Born-closure correction. See the text for the further discussion.

### Positron Scattering

The integral cross-sectional results for positron scattering are shown in Figure 4 in the SP and SP+Born levels of approximation and compared to the SEP+Born calculation for electron scattering. Describing the polarization of the target correctly in positron scattering is more difficult than that for electrons. The static positron-molecule potential is repulsive, while the polarization potential is attractive. Therefore, an accurate description of the distortion of the electronic cloud is crucial. As in the case of electron scattering, there are no experimental or theoretical data for positron collisions with lysine; therefore, it is challenging to determine whether the description of polarization adopted in this work was adequate. Therefore, we performed the best calculation possible within the limits of our computational resources[Fn fn2]. Additionally, the formation of a positronium channel is not explicitly considered in our calculations, so we do not expect good agreement for energies above the threshold (*E*_*Ps*_=*IP*–6.8;(eV)), which in our calculation is estimated at 3.77 eV for lysine.

**Figure 4:**
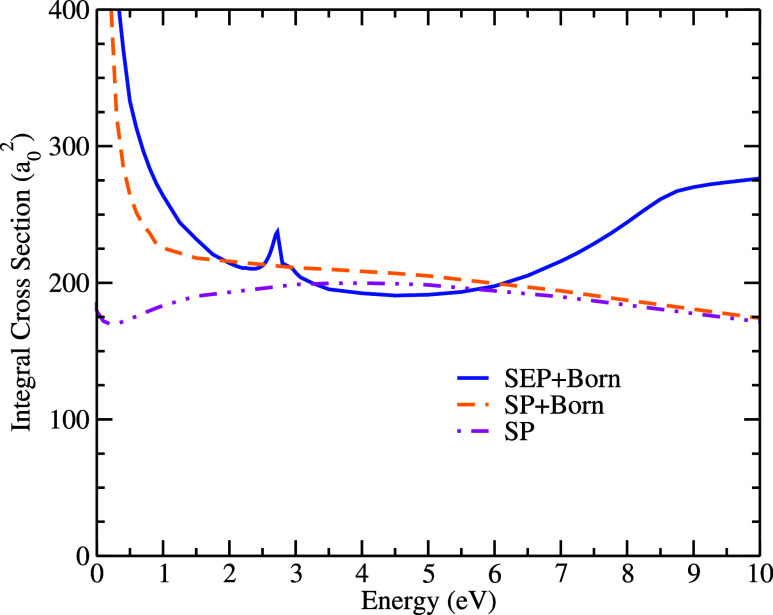
Elastic integral cross sections for positron scattering with the lysine molecule, obtained at the SP and SP+Born levels of approximation. For comparison, the electron scattering result at the SEP+Born level is also shown.

The comparison between electron (SEP+Born) and positron (SP+Born) cross sections shows that both curves exhibit a similar shape at very low energies, with the electrons being higher in magnitude. As the impact energy increases (between 4 and 6 eV), the cross sections become very close in magnitude but differ in shape for the two projectiles. Above 6 eV, the cross section obtained for electron scattering has both shape and magnitude that are very different from those obtained for the case of positron scattering. This difference between the two results is mainly due to the resonant structures present in the electron cross section in this range of energy, as discussed previously. However, it is necessary to remember that above 3.77 eV we have the formation of the positronium channel, meaning that the elastic cross section for positrons may be overestimated since part of the process that would go to the positronium formation channel is not considered in our calculations.

The differential cross section for positron scattering is shown in Figure 5. As in the case for electrons, for higher angles (typically above 20°), there is good agreement between the DCS results with and without Born approximation. Also, for 3 eV, we observed the *p*-type, *d*-type for 5 and 7 eV, and *f*-type for 10 eV. Comparing the DCSs for electron and positron, we can see that, as energy increases, the magnitude of the electron DCSs becomes greater than those obtained in positron scattering, particularly for angles above 40 °. However, the wave patterns of DCSs are the same for both projectiles.

**Figure 5:**
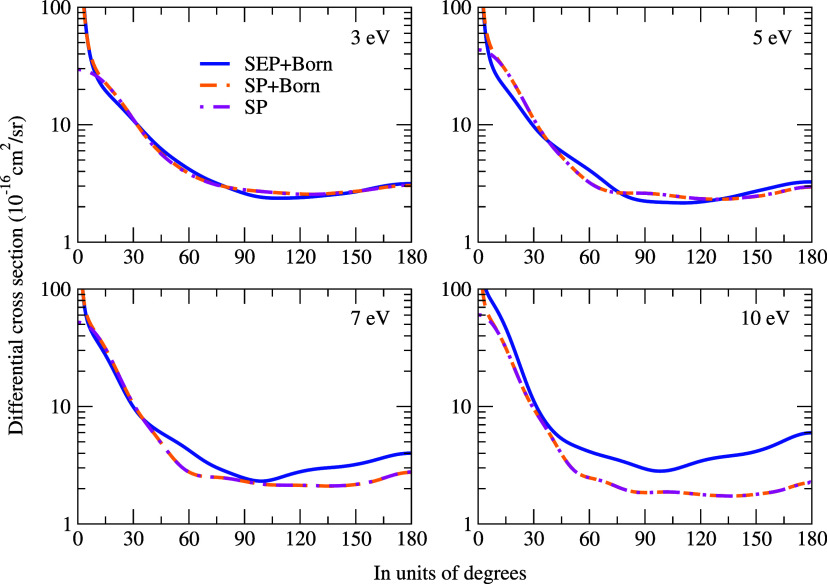
Differential cross sections for positron and electron (SEP+Born) scattering for the lysine molecule. We compare our results obtained at the SP approximation with and without Born-closure correction. See the text for the further discussion.

### DEA Thresholds for Hydrogen Loss

To investigate the possibility of π* resonance being able to perform DEA, we have conducted additional electronic structure calculations to estimate threshold energies for hydrogen loss, mediated by DEA. These threshold energies (*E*_*th*_) were obtained as *E*_*th*_ = *E*_*A* ^–^_ + *E*_*B*_ – *E*_0_, where *E*_*A* ^–^_ is the energy of the anionic fragment, *E*_*B*_ is the energy of the neutral fragment, and *E*_0_ is the energy of the parent molecule. We performed these calculations for hydrogen atoms, labeled from A to F (Figure 1), and the results are displayed in Table 6. These calculations were carried out in the B3LYP/aug-cc-pVDZ level of approximation. The analysis reveals that the hydrogen loss from the oxygen atom exhibits a low barrier of only 1.85 eV for hydrogen H_*A*_, closely aligning with the estimated result of the resonance position obtained through VAE. This finding could suggest that this pathway is a viable route for DEA in lysine, since the π* resonance is located mostly at C=O double bond, which is close to the H_*A*_ hydrogen. For all other hydrogens, the energy required is much higher and other processes must be considered for dissociation. The main reason to estimate the threshold for this channel is due to the fact that previous DEA studies with amino acids^19–23^ have shown that the most intense channel, at around 1–2 eV, is due to hydrogen loss. Moreover, these studies, in general, indicate that the H loss occurs in the carboxylic group of the amino acids, which is in close agreement with our results. For a better comparison, in Table 5, we list the values found in the literature for resonance and H-loss threshold for the cited amino acids, including our calculations for lysine. Regarding the choice of selected hydrogens: we tried to choose H atoms along the molecule. Not only those closer to the π* resonance region but also others in other sites of the molecule. Similar calculations for the other H atoms indicate energy thresholds of next to 4 eV.

**Table 5:** Resonance Energy π* and DEA Threshold (H-Loss) for the Selected Amino Acids

amino acid	π* (eV)	*E*_DEA_ (eV)
glycine^19^	1.93	1.18–1.27
alanine^19^	1.80	1.18–1.27
proline^19,21^	1.91	1.25–1.49
lysine (this work)	1.65	1.85

To further investigate this possibility, we diagonalized the H_*N*+1_ Hamiltonian and identified the eigenstate corresponding to the resonant structure observed in the cross sections. The graph of this function is shown in Figure 6. Gallup^39^ showed that, in formic acid, a short-lived σ* resonance located in the O*-*H bond is directly responsible for the loss of hydrogen. This was later confirmed in an experiment by Allan^40^ in formic acid, in contrast to a previous theoretical prediction of an indirect dissociation.^41^ However, as can be seen in Figure 6, the graph does not show significant electron density in the O*-*H_*A*_ bond for the lysine molecule. Therefore, based on our results, we suggest that, at low energy, DEA occurs predominantly through the indirect pathway associated with the presence of π*, but further investigations are needed. Therefore, we now analyze electronically excited states and their potential role in the DEA mechanism, as discussed in the Section Electronic Excited States and Implications for DEA (Table 6).

**Figure 6:**
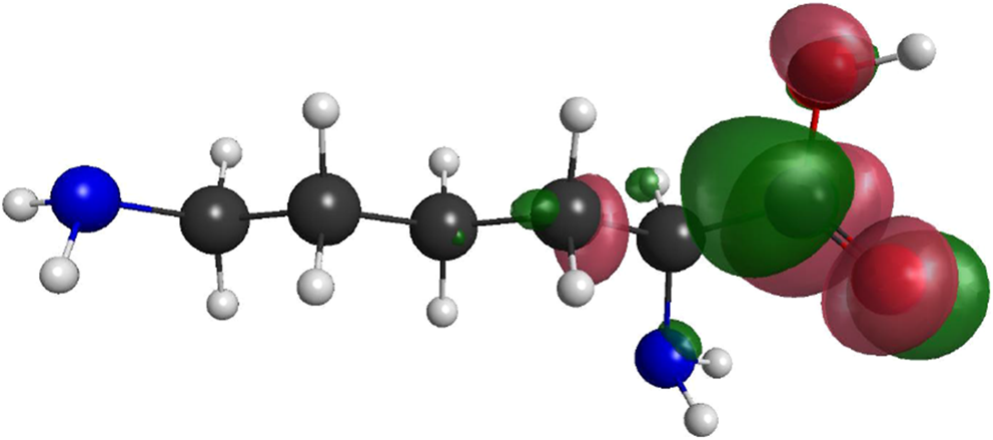
Resonant orbital (π_C=O_*) of lysine obtained from the diagonalization of the (*N*+1) -electron Hamiltonian. The figure shows the spatial form of the resonant wave function.

**Table 6:** Thresholds for H Loss, Obtained as the Sum of Anionic (Lysine-H_*i*_) and Neutral (H_*i*_) Fragment Energies, Minus the Parent Molecule Energy^a^

	*E*_*th*_ (eV)
H_*A*_	1.85
H_*B*_	3.47
H_*C*_	3.72
H_*D*_	3.60
H_*E*_	4.26
H_*F*_	4.40

a The calculations were carried out at the B3LYP/aug-cc-pVDZ level of approximation.

### Electronic Excited States and Implications for DEA

To complement the analysis of the DEA process, we investigated the electronic excited states of the lysine. These states may act as parent states in DEA processes mediated by Feshbach or core-excited resonances, providing an additional pathway for molecular fragmentation upon electron or positron impact. The calculations were performed using GAMESS^27^ within the time-dependent density functional theory (TDDFT) with the B3LYP functional and aug-cc-pVDZ basis set to obtain the energy of the excited states of lysine and molecular orbitals that have the most important contributions in the description. Such studies are not intended as a comprehensive treatment of electronically excited states but rather as supportive results that may guide future investigations of electron- and positron-induced excitation and fragmentation.

The dominant molecular (hole and particle) orbitals are in Figure 7 and the energies for the first five excited states (three triplets and two singlets) *+* are displayed in Table 7. Additionally, the pairs involved in the excitations ([hole→particle]) and their respective contributions to the description of each excited state are also shown. For instance, the low-lying triplet electronic excited state is at 4.972 eV, and it is described accurately (with 95%) using four hole-particle pairs, as we can see in Table 7. This is important since further studies on electronic excitation by positron or electron impact requires the correct description of these states. Interestingly, the energies of these low-lying states are close to the H-loss thresholds given in Table 6, suggesting that they could play a role in Feshbach-type DEA channels. However, we emphasize that this assignment remains tentative. Additional studies combining higher-level electronic structure methods (including calculations along selected reactive coordinates) and experimental data will be needed for definitive identification. In this sense, the present results should be seen as an initial step that provides a useful framework for follow-up investigations, not only for lysine but also for other similar biomolecules.

**Figure 7:**
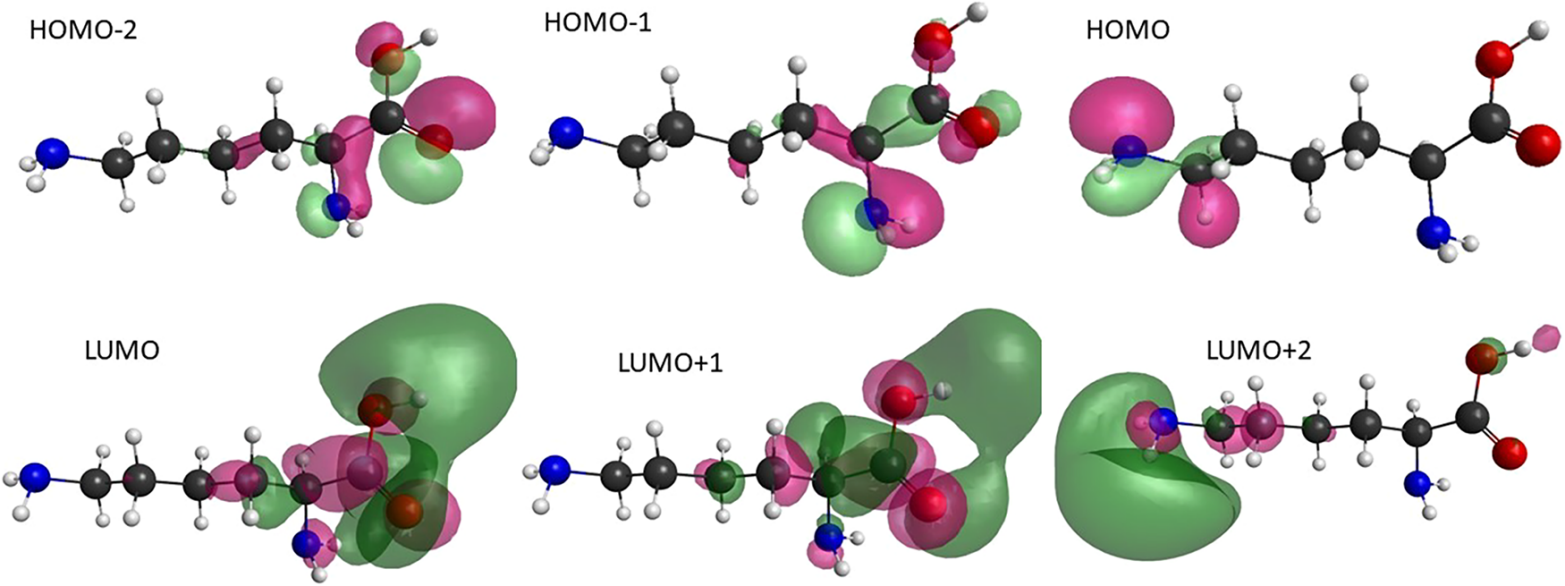
Molecular orbitals with the greatest contribution to the description of the first five excited states of the lysine molecule. Upper line: the highest (HOMO), the second highest (HOMO–1) and the third highest (HOMO–2) occupied molecular orbitals. Lower line: lowest (LUMO), the second-lowest (LUMO+1), and the third-lowest (LUMO+2) unoccupied molecular orbitals.

**Table 7:** Calculated Dominant Vertical Excitation Energies (TDDFT/B3LYP/aug-cc-pVDZ)^a^

state	*E* (eV)	dominant excitations (contribution)
1 ^3^ *A*	4.972	HOMO–1 → LUMO+1 (27%) + HOMO–1 → LUMO (27%) + HOMO–2 → LUMO+1 (22%) + HOMO–2 → LUMO (19%)
2 ^3^ *A*	5.185	HOMO → LUMO+2 (85%)
1 ^1^ *A*	5.369	HOMO → LUMO+2 (87%)
2 ^1^ *A*	5.456	HOMO–1 → LUMO (44%) + HOMO–1 → LUMO+1 (33%) + HOMO–2 → LUMO+1 (11%)
3 ^3^ *A*	5.537	HOMO–1 → LUMO (30%) + HOMO–2 → LUMO+1 (24%) + HOMO–2 → LUMO+1 (21%)

a See the text for the further discussion.

## Conclusions

We have carried out a theoretical investigation of elastic low-energy electron and positron scattering by the lysine molecule, aiming to provide cross-sectional data and identify possible resonant and dissociative features relevant to biological environments.

For electron scattering, we identified π*-type resonances at 3.95 eV (SE) and 2.73 eV (SEP). Additionally, using a semiempirical scaling relation, we estimated a vertical attachment energy of 1.65 eV. Complementary electronic structure calculations revealed a threshold of 1.85 eV for hydrogen loss near the carboxylic group, suggesting that DEA may occur via this resonant channel. These findings are consistent with those of previous studies on other amino acids. Moreover, we found a σ*resonances overlap around 12.2 eV (SE) and 10.5 eV (SEP). We emphasize that several of our analyses rely on VAE estimates obtained from semiempirical relationships based on Koopmans’ theorem. Although approximate and subject to uncertainty, this approach is validated in the literature and provides a consistent guideline. In our case, the inclusion of polarization shifts the resonance toward the VAE, suggesting that larger configuration spaces could yield even closer agreement, which was not possible in our case due to the computational limit. Because of these issues, although the method is not exact, both our results and those reported in the literature for other amino acids support the use of the VAE as a basis for our discussion. Furthermore, as shown in the DEA Thresholds for Hydrogen Loss section, the H-loss values are sufficiently distant from each other to allow us to use the VAE value, which, even if the estimate is not exact, provides a good guide to the importance of the π* resonance.

We also analyzed the angular and integral cross sections for both projectiles. Although the dominant angular patterns in the differential cross sections are similar for electrons and positrons, the cross sections for electrons are significantly larger, especially above 6 eV, where the resonance structures become prominent. The integral cross sections exhibit similar behavior only at very low energies, primarily due to the long-range interaction with the molecular dipole moment.

To complement the interpretation of possible DEA pathways and investigate the possibility that the π* resonance found is related to this process, we calculated thresholds for hydrogen loss. Although the energy found for the loss of *H*_*A*_ is close to the VAE value, suggesting that this resonance occurring in the C = O bond is directly responsible for this loss, the same conclusion cannot be drawn by directly examining the resonant orbital. This does not mean that this resonance cannot participate in the *H*_*A*_ loss process, but rather that, if it does participate, it is not through a direct process. Thus, to gain some insight into the origin of the DEA process in lysine, we calculated the first few electronic excited states of lysine using TDDFT. These excited states lie near the calculated dissociation thresholds and may act as parent states for Feshbach or core-excited resonances, which could contribute to alternative DEA mechanisms. Although not a fully conclusive result, this is a first result that should be further investigated. Furthermore, it highlights the importance of including excitation channels in future scattering models.

Overall, this study provides the first theoretical insights into electron and positron scattering by lysine and points to the need for further theoretical and experimental work, including inelastic scattering and direct DEA cross-sectional measurements.

## Footnotes

a Calculated data for the cross section can be found at https://github.com/cammaral/lysine-smc

b We recall that lysine has a total of 104 quantum subsystems (24 nuclei +80 electrons). Our calculations were performed in clusters at the Universidade Federal do Paraná.
